# Higher prevalence of incidental findings identified upon coronary calcium score assessment in type 2 and type 3 diabetes versus type 1 diabetes

**DOI:** 10.1371/journal.pone.0251693

**Published:** 2021-05-24

**Authors:** Mélanie Gaudillière, Charlotte Marsot, Laetitia Balaire, Laure Groisne, Myriam Moret, Sylvie Villar-Fimbel, Philippe Douek, Philippe Moulin, Sybil Charrière

**Affiliations:** 1 Faculté de médecine Lyon EST, Université Lyon 1 Claude Bernard, Lyon, France; 2 Hospices Civils de Lyon, Hôpital Cardiovasculaire Louis Pradel, Fédération d’endocrinologie, maladies métaboliques, diabète et nutrition, Bron cedex, France; 3 Hospices Civils de Lyon, Service de radiologie, Hôpital Louis Pradel, Bron cedex, France; 4 Laboratoire Creatis, CNRS UMR 5220, INSERM U12063, Université Claude Bernard Lyon 1, Villeurbanne, France; 5 INSERM U1060, Laboratoire Carmen, Université Lyon 1, INRA U1235, INSA de Lyon, CENS, Centre de Recherche en Nutrition Humaine Rhône Alpes, Villeurbanne, Oullins cedex, France; Medizinische Universitat Graz, AUSTRIA

## Abstract

**Aim:**

Noninvasive assessment of infraclinic coronary atherosclerosis by coronary artery calcium score (CAC) measurement leads to the identification of incidental findings. The aim of this study was to determine the prevalence of incidental findings following systematic CAC assessment in diabetic patients with high cardiovascular risk, to identify the determinants, and to assess the midterm consequences of these findings in patient care.

**Methods:**

732 consecutive asymptomatic patients (187 type 1 diabetes (TD1), 482 type 2 diabetes (TD2) and 63 type 3 diabetes (TD3)) aged 60.6±0.7 years who had a CAC assessment by Multiple Detector Computed Tomography between 2015 and 2017 were systematically included. Clinical and biological data were collected from medical electronic files.

**Results:**

117/732 diabetic patients (16.0%) had incidental findings of which 105 (14.3%) were unknown. Incidental findings were more frequent in TD3 (23.8%) and TD2 (17.0%) than in TD1 (10.7%) (p = 0.05). 76 diabetic patients (10.4%) had lung abnormalities, mainly pulmonary nodules (31 patients, 4.2%). The other incidental finding were pericardial (1.5%), vascular (1.2%), thymic (0.7%) and digestive diseases (0.5%). 42.6% of patients with incidental findings had an additional TDM and 56.8% a specialized medical advice. In 10 patients (9.3% of incidental findings), the identification of incidental finding led to a specific treatment of the underlying disease. In multivariate analysis, microalbuminuria, type of diabetes (TD2/TD3 vs TD1) and smoking were significantly associated with incidental findings (p = 0.003; p = 0.026; p = 0.050 respectively).

**Conclusions:**

Incidental findings are not rare in diabetic patients upon CAC assessment. A fraction of them are accessible to specific treatment. These findings raise the question if a systematic low dose chest TDM should be conducted in TD2 or TD3 patients and in any diabetic smokers by enlarging the window used for CAC assessment.

## Introduction

Diabetes is a major risk factor of ischemic heart diseases. Severe coronary atherosclerosis is often silent in diabetic patients [1]. Consequently, it has been proposed to screen diabetic patients for infraclinic coronary atherosclerosis by calcium scoring in order to conduct functional testing only in the specific subgroup of high risk patients with severe infraclinic calcified coronary atherosclerosis [2–8]. Thus, Coronary Artery Calcium Score (CAC) measurement by Multiple Detector Computed Tomography (MDCT) is nowadays recommended to measure the intensity and the diffusion of silent coronary calcified plaques, to reclassify cardiovascular risk by several scientific societies [9,10].

The field of view upon CAC assessment includes the heart, the mediastinum, the upper part of the liver and the central part of the lungs. Thus, incidental findings of thoracic and abdominal lesions, within the window used for CAC, may be of interest when setting recommendations regarding the systematic screening of infraclinic coronary atherosclerosis in high risk diabetic patients [11–13]. Identification of incidental findings may provide a unique opportunity to detect neoplasic lesions which could be cured only at an early asymptomatic stage. Recently, the US Preventive Services Task Force recommended to conduct studies in real life which would explore the yield of incidental findings detection following a CAC assessment and the downstream consequences [12]. Thus, we conducted a retrospective systematic study of 732 consecutive asymptomatic diabetic subjects at high cardiovascular (CV) risk who underwent a CAC assessment (DISCO cohort). The aims of this study were to evaluate the prevalence of any incidental findings discovered, to identify the predicting factors and finally to report the midterm consequences of these findings in medical care.

## Materials and methods

### Study design

A retrospective descriptive monocentric study (DISCO cohort) was performed in the Diabetology department of Louis Pradel Cardiovascular Hospital, in Lyon (France). All diabetic patients who had a CAC assessment between 01 January 2015 and 31 December 2016 were systematically included. CAC assessment was added in our routine practice since 2013 to improve cardiovascular risk estimation in asymptomatic diabetic patients over 40 years old in primary prevention, in order to conduct a more personalized prevention and to identify very high risk patients eligible for detection of silent myocardial ischemia, as now recommended by current guidelines [9,10].

The downstream clinical consequences were monitored for the next 2 years (23 months following the last CAC performed). Incidental findings were considered when the patients had no history of the suspected disease in their medical records. Sex, age, Body Mass Index (BMI), type of diabetes (type 1 (TD1), type 2 (TD2) or type 3 (TD3)), diabetes duration, HbA1c, CAC value, presence of hypertension treated or not, retinopathy and nephropathy (microalbuminuria between 20–200 mg/ml, renal failure measured by creatinemia and CKD formula <60ml/min/m2), treatment by insulin and smoking quantified in Pack-Years (PY), active or weaned, were collected in electronic medical records, with a high completeness (98% of data collected).

Type 3 diabetic patients included secondary diabetes (post pancreatic surgery, hemochromatosis, post pancreatitis, pancreatic neoplasia), and monogenic diabetes.

Data were fully anonymized before statistical analysis. The patient’s medical records were accessed between January 2018 and December 2018.

This study was performed after agreement of the ethics committee of our hospital (Hospices Civils de Lyon, N°19–111). The database was declared to the national data protection committee (Commission nationale de l’informatique et des libertés, N°19–234.) and all the patients received an information notice about the study in order to collect their written consent, in agreement with the legislation in place at the time of the study (French bioethics law Jardé).

### CAC assessment

A MDCT Brillance 64 (Philips Healthcare) was used for data acquisition. The scanning protocol acquired images prospectively (pitch N/A) with ECG-triggering, at 120 Kvp, appropriate mAs adapted to the patient size (80–160 mAs) with a 0.33- millisecond gantry rotation time, an individual detector width of 64 mm with a reconstructed section width of 2.5 mm, and temporal resolution of 0.33 milliseconds. Contiguous 0.9 mm-thick sections were reconstructed using iterative reconstruction (I dose level 3) half-scan interpolation from the left mainstem bronchus to the cardiac apex during peak inspiration with a 25 cm field of view. Agatston scores were quantified on CT Philips workstations (Heartbeat-CS, Philips Healthcare) and were expressed in Agatston units (AU). The obtained images encompassed some parts of the lungs (one half to two-thirds), the superior one-third of the liver, the superior one- quarter of the spleen, the mediastinum, and the inferior one-quarter of the trachea. Incidental findings, as described by radiologists from radiology reports, were recorded. No rereading was performed.

### Statistical analysis

Statistical analysis was performed using SPSS software (version 20.0, SPSS Inc., Chicago, IL). The level of significance was 2 sided and set at 5% (p<0.05). To test the normal distribution of quantitative variables, a Shapiro-Wilk’s test was used (p>0.05). All variables, with the exception of coronary artery score, were normally distributed. Quantitative variables are expressed as mean ± standard error (SE) and categorical variables were expressed as number (n) and percentage. When the distribution was not normal, quantitative variables were expressed as median and interquartile range [IQR]. To compare subject characteristics and MDCT data, Chi2 test or Fisher’s exact tests were used for categorical variables and ANOVA for quantitative variables. When the distribution was not normal, a Wilcoxon-Mann- Whitney test was used to compare the median of quantitative variables. To determine independent predictors of incidental findings or pulmonary nodules, a multivariate analysis using a binary logistic regression model (entry method) was performed with variables with p<0.05 in univariate analysis, and relevant baseline characteristics.

## Results

### Clinical characteristics of the subjects

732 diabetic patients were included. Patient characteristics are summarized in Table 1. They were middle aged, with a balanced sex ratio and a mild obesity. They had a long duration of diabetes: 40.2% had more than 20 years of diabetes duration. Most of the patients had TD2. They had and on average 2.1 cardiovascular risk factors and poor glycemic control. More than 40% had at least one microvascular complication. The median CAC was 29 AU [IQR 227]. 65% of the patients had a CAC value below 100. The distribution of type of diabetes among CAC groups (< 100, 100–400, > 400) was not statistically different (p = 0.510) (S1 Table).

**Table 1:** Clinical characteristics of the patients.

	n = 732
**Sex (male)**	399 (54.5)
**Age (years)**	60.6 (+/- 0.4)
**BMI (kg/m2)**	29.6 (+/-0.2)
**Diabetes duration (years)**	18.4 (+/- 0.1)
**Less than 20 years**	438 (59.8)
**More than 20 years**	294 (40.2)
**HBA1c (%)**	8.46 (+/- 0.07)
**CAC value (AU)**	29 [227]
**Type of diabetes**	
**Type 1 diabetes**	187 (25.5)
**Type 2 diabetes**	482 (65.8)
**Type 3 diabetes**	63 (8.6)
**Insulin**	411 (56.1)
**Smoking habits**	
**Never smoking**	415 (56.7)
**Active smoking**	128 (17.5)
**Weaned < 3 years**	32 (4.4)
**Weaned > 3 years**	157 (21.4)
**Hypertension**	461 (63)
**Retinopathy (n = 726)**	307 (42.3)
**Nephropathy**	
**Microalbuminuria (20–200 mg/l)**	264 (36.1)
**Renal failure (<60ml/min/m2)**	76 (10.4)

Categorical variables ie sex, type of diabetes, presence of incidental findings, insulin therapy, smoking, hypertension, retinopathy and nephropathy are expressed in number (percentage). Quantitative variables are expressed as mean (± standard error of the mean (SEM)). CAC is expressed as median [IQR].

AU: Agaston unit; PY: Pack-years.

### Incidental findings

**Fig 1:**
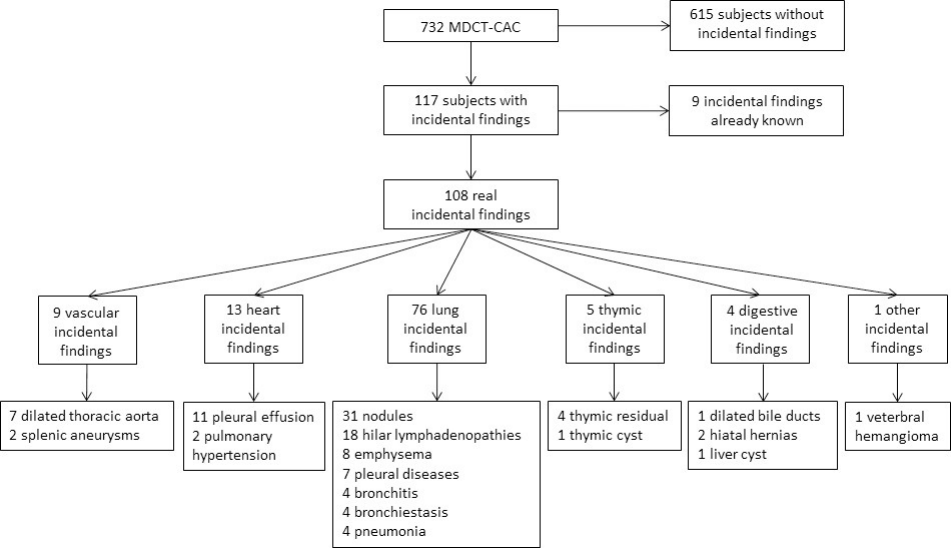
Breakdown of incidental findings. MDCT-CAC: Multiple Detector Computed Tomography—Coronary Artery Calcium Score.

**Fig 2:**
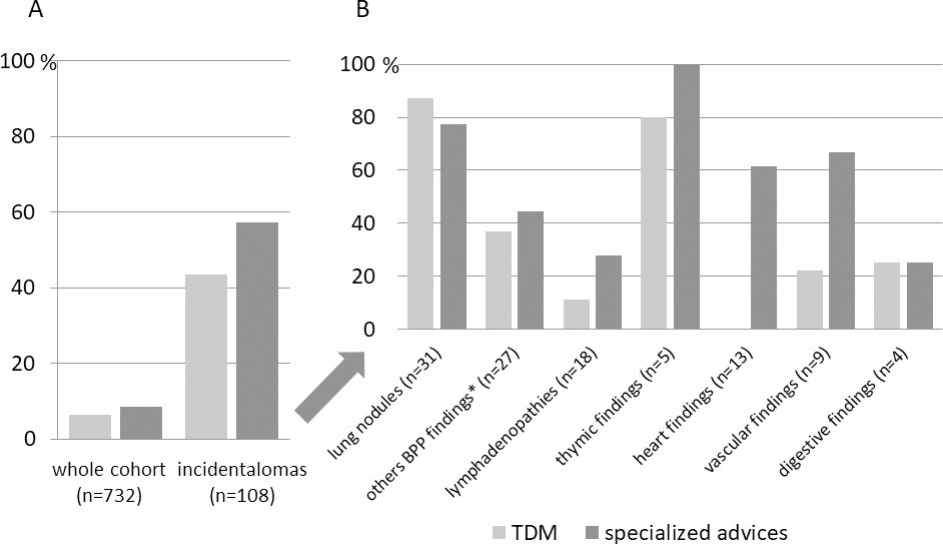
Tomodensitometry and specialized advices following incidental findings discovery upon CAC assessement. Results are presented over the whole cohort and all incidental findings (A) and splitted by type of incidental findings (B). *BPP: Bronchopulmonary and pleural, † no TDM realized but 6 cardiac echographies.

The breakdown of incidental findings is shown in the Fig 1. 16.0% of the diabetic patients had incidental findings including 1.3% related to the patient past history. Incidental findings were more frequent in type 3 diabetes (n = 15/63, 23.8%) and in type 2 diabetes (n = 82/482, 17.0%) than in type 1 diabetes (n = 20/187, 10.7%) (p = 0.041 for type 2 vs type 1, p = 0.009 for type 3 vs type 1, p = 0.185 for type 2 vs type 3). Only 12 incidental findings were not taken into account by the diabetologist in charge of the patient without any clear reason identified in medical reports. Eighty four patients with newly found incidental findings (77.8%) underwent additional investigations coordinated by the diabetologist in charge of the patient. The percentage of additional tomodensitometry (TDM) and specialized medical advices following the incidental findings discovery are summarized in the Fig 2.

#### Lung incidental findings

Most of the incidental findings were pulmonary (70.4%). 10.4% (76/732) of these asymptomatic diabetic patients presented a lung incidental finding: 31/76 had nodules, 18 hilar lymphadenopathies, 8 emphysema, 7 pleural diseases, 4 bronchiectasis, 4 bronchitis and 4 pneumonia.

Among the 31 patients with lung nodules, 38.7% had micronodules with a size less than 5 mm, 35.5% had nodules 5–10 mm, 19.4% had nodules 10–30 mm and 2 patients had a nodule size above 30 mm. The diagnosis and the therapeutic management of patients with lung nodules are detailed in the S2 Table. 87% of patients had a thoracic TDM and 77% a specialized advice. None of the micronodules had significantly increased or had become cancerous, over an average 23 months follow-up. Three patients with nodules ≥ 30 mm,had pulmonary carcinoma (S1 File and S3 Table).

The medical cares regarding the other pulmonary incidental findings are summarized in the S4 Table.

#### Others incidental findings

After lung incidentals findings, heart and vascular incidentals findings were the most frequent (12.0% and 8.3%, respectively). The details and the medical cares regarding heart, vascular, thymic, digestive and vertebral incindental findings are summarized in the S5 Table.

To summarize, 42.6% of patients with incidental findings had a TDM and 56.8% a specialized medical evaluation representing respectively 6.3% and 8.3% of the whole cohort (Fig 2A). For 10 patients (9.3% of incidental findings or 1.4% of all patients), the identification of incidental findings led to a specific treatment of the underlying disease.

### Characteristics of diabetic patients with and without any incidental findings

No significant differences regarding sex, age, BMI and diabetes duration between diabetic patients with and without incidental findings were found (Table 2). Incidental findings were more often found in patients who smoked more than 20 pack-years (PY) (26.5% vs 16.5% for less than 20 PY and 14.2% for those who never smoked; p = 0.040). No significant difference regarding active smoking, weaned smoking for less than 3 years or more than 3 years was found. Incidental findings were more often found in patients with a type 3 diabetes (23.8% vs 10.7% for type 1 diabetes and 17.0% for type 2 diabetes, p = 0.028). Blood glucose control and need for insulin was similar between both groups of diabetic patients. CAC, hypertension prevalence and blood pressure levels were similar in both groups. However, diabetic patients with nephropathy had more incidental findings considering either microalbuminuria (21.2% vs 13%; p = 0.004) or renal failure (GFR < 60 ml/mn, 23.7% vs 15.1%; p = 0.053) whereas it was not the case for patients with retinopathy.

**Table 2:** Characteristics of subjects with or without incidental findings and pulmonary nodules.

	Incidental findings (n = 117)	No incidental findings (n = 615)	p	Nodules (n = 31)	No nodules (n = 701)	p
**Sex (male)**	60 (51.3)	339 (55.1)	0.445	21 (67.7)	378 (53.9)	0.131
**Age (years)**	62.1 (+/-1.0)	60.3 (+/-0.4)	0.075	61.3 (+/- 1.4)	60.6 (+/- 0.4)	0.664
**BMI (kg/m2)**	29.5 (+/-0.6)	29.7 (+/-0.2)	0.744	29.2 (+/-0.8)	29.7 (+/-0.2)	0.655
**Diabetes duration (years)**	17.71 (+/-0.96)	18.57 (+/-0.45)	0.438	16.5 (+/-1.6)	18.5 (+/-0.4)	0.322
**HbA1c**	8.25 (+/-0.16)	8.51 (+/-0.08)	0.180	8.42 (+/-0.34)	8.47 (+/-0.07)	0.893
**CAC (AU)**	29 [298]	29 [213]	0.854	41 [749]	29 [225]	0.854
**Type of diabetes**						
**Type 1**	20 (17.1)	167 (27.2)		3 (9.7)	184 (26.2)	
**Type 2**	82 (70.1)	400 (65)	0.028	22 (71.0)	460 (65.6)	0.022
**Type 3**	15 (12.8)	48 (7.8)		6 (19.3)	57 (8.1)	
**Insulin therapy**	62 (53.0)	349 (56.7)	0.453	14 (45.2)	397 (56.6)	0.208
**Smoking**						
**Never**	59 (50.4)	356 (57.9)	0.136	12 (38.7)	403 (57.5)	0.039
**Active or weaned**	58 (49.6)	259 (42.1)		19 (61.3)	298 (42.5)	
**Hypertension**	77 (65.8)	384 (62.4)	0.489	16 (51.6)	445 (63.5)	0.181
**Retinopathy(n = 726)**	51 (43.6)	256 (42.0)	0.755	9 (29.0)	298 (42.9)	0.127
Micro-albuminuria*	56 (47.9)	208 (33.8)	0.004	17 (54.8)	247 (35.2)	0.026
**Renal failure†**	18 (15.4)	58 (9.4)	0.053	4 (12.9)	72 (10.3)	0.638

Categorical variables are expressed in number (percentage). Quantitative variables are expressed as mean ± standard error of the mean (SEM) CAC is expressed as median [IQR].

*Microalbuminuria: 20–200 mg/l.

†Renal failure: <60ml/min/m2 AU: Agaston unit.

In multivariate analysis (following adjustment on age, sex, BMI, diabetes duration, HbA1c, and renal failure), microalbuminuria, type of diabetes (TD2 and TD3 vs TD1) and smoking (active or weaned vs never) remained independently associated with incidental findings (respectively OR 1.89(1.23–2.90), p = 0.003; OR 1.99(1.09–3.67), p = 0.026; OR 1.56 (1.00–2.42), p = 0.050).

### Characteristics of subjects with and without pulmonary nodules

No significant differences regarding sex, age, BMI and diabetes duration between subjects with and without pulmonary nodules were found (Table 2). Again, nodules prevalence was higher in patients with a type 3 diabetes (9.5% vs 1.6% for type 1 diabetes and 4.6% for type 2 diabetes, p = 0.022), and in patients with microalbuminuria (6.4% vs 3%, p = 0.026). Active or weaned smokers also had more pulmonary nodules (p = 0.039).

In multivariate analysis following adjustment on age, sex, BMI, diabetes duration, HbA1c, and renal failure, both microalbuminuria and type of diabetes (TD2 and TD3 vs TD1) remained independently associated with pulmonary nodules (OR 2.19 (1.02–4.68), p = 0.043 and OR 4.74 (1.23–18.22), p = 0.024, respectively), but not smoking (p = 0.127).

## Discussion

This study reports a high rate of incidental findings in our diabetic population since incidental findings were found in about one out of six patients, including mostly pulmonary findings (70.4%) and of which 28.7% were lung nodules. The exact prevalence of lung incidental findings in these patients is likely to be underestimated since the window used for the CAC assessment does not take in account the upper third of the chest.

Other studies in the general population have considered the prevalence of incidental findings found upon CAC assessment. They report a highly variable prevalence, ranging from about 8% to 25% [13,14,16,17] and even over 50% [12,18,19]. Few early studies involved Electron Beam Tomography (EBT) instead of MDCT but this did not substantially affect the yield of incidental findings detection [13–16]. Conversely, the heterogeneity of the studied populations might explain the variable prevalence. The percentage of current or former smokers is highly variable (respectively 6.7 to 42% and 18.3 to 39%) [8,14–19]. The percentage of diabetic patients is unknown in some cohort [13,15,19]; diabetics patients were excluded in the study of Haller et al. [17] and only 18% were included in the cohort of Onuma et al. [18]. In this last study, there is no report of a higher prevalence of incidental findings in diabetic patients. A systematic description of all incidental findings revealed in a diabetic population and their management was not performed in any study.

The higher prevalence of incidental findings in type 2 diabetes than in type 1 diabetes may be related to general insulin resistance and inflammation involved in the pathophysiology of type 2 diabetes, whereas type 1 diabetes is a pancreatic auto-immune disease. An increased cancer prevalence has been reported in both in type 2 diabetes and metabolic syndrome. The reported cancers were endometrial, breast, lung, pancreatic, hepatocellular and colorectal cancers [20,21]. Altough insulin has been suspected to be involved in a higher incidence of cancer by inducing an overexpression of growth factors, no association with the prevalence of incidental findings was found.

However, we also found in our cohort a higher occurrence of incidental findings in type 3 diabetes. These patients are more often in frailty conditions and may exhibits additional comorbidities contributing indirectly to the increased prevalence of incidental findings we found. The difference of incidental findings between type 3 and type 2 diabetes was not significant, probably by a lack of power with a small type 3 diabetes group. Nevertheless, only three lung cancers were identified in our cohort, all in type 2 diabetic patients. Lung cancer prevalence ranged from 0–1.2% in previous studies [13–19] leading to an overall prevalence of 0.18% which is not significantly lower than that found in our cohort (0.4%).

Unsurprisingly, active or weaned smoking is associated to incidental findings in our cohort. Most incidental findings identified are pulmonary and smoking is a risk factor for a variety of lung diseases apart lung cancers (chronic obstructive bronchitis, emphysema, fibrosis, bronchiectasis …) [22]. The association of microalbuminuria with both incidental findings and pulmonary nodules is unexpected and was not previously documented in literature. Nevertheless, microalbuminuria and diabetic nephropathy are associated with an activation of fibrosis, oxidative stress and chronic inflammation pathways, also involved in tumorigenesis [23,24]. Further investigations are needed to confirm and/or elucidate the potential link between micro-albuminuria and incidental findings in diabetic patients.

Regarding the care monitored in real life, almost half of the patients with incidental findings had an additional TDM, leading to an increased irradiation. However, this represents only 6.3% of the whole cohort. Recent studies reported a reduction of lung cancer mortality after regular screening by low-dose computed tomography (LDCT) in heavy smokers [25,26]. Since smoking is a common risk factor for both ischemic coronary disease and lung cancer risk, the question of performing a LDCT directly coupled with CAC in smokers and/or in patients with a higher cancer risk such as type 2 diabetic patients is open. Moreover, LDCT would increase the sensibility of detection of lung nodules and parenchymal lung abnormalities compared to MCDT performed for CAC assessment. Conversely, such a strategy could increase lung cancer overdiagnosis: a recent meta-analysis considered almost half of lung cancers detected by LDCT were over diagnosed [27].

In our study, the CAC and the subsequent TDM allowed an early diagnosis of lung cancer in three smoking patients who were still alive after two and three years of follow-up. Among the micronodules, none of them had significantly increased or became cancerous. However the duration of follow-up (on average 23 months) was too short in this study to determine whether this careful monitoring will be beneficial to these patients on a long term follow up.

Thus, the tradeoff between risks and benefits is important to consider in this population with a high CV risk. The benefit of an early identification of an incidental finding using MDCT provides a unique opportunity to detect an illness early- phase for which the outcome could be improved by early treatment. Conversely, several studies raise the issue of insignificant accidental discoveries, which in addition to anxiety, would lead to further exploration increasing irradiation without proof of a real benefit [12].

One limitation of our study is the monocentric design leading to a limited number of patients in the cohort, which makes statistical analysis less powerful. Additionally, the lack of rereading performed to check for oversights could be a second limitation. However most of the incidental findings were submitted to an independent specialized expert. Their consistency was established, and this situation corresponds to real life.

Furthermore, the duration of lung nodules follow-up was short because they require a demonstration of stability over at least 2 years and our study was only conducted during 23 months. The retrospective design precludes any bias of observation regarding the evaluation of the efficiency of care provided following CAC assessment. It represents a fair description of the care based on the electronic medical file with an excellent completeness since 98% of the items were collected in database. Few data is available in literature on the yield of the downstream care following CAC assessment.

## Conclusions

To conclude, our findings raise the question of a need to combine CAC assessment with a LDCT, in order to explore a whole chest window with more sensibility, in type 2 and type 3 diabetic patients or in smokers, who are most likely to present incidental findings or cancers. Additional cohort studies comparing different options are needed to validate such a strategy.

## Supporting information

S1 Table Distribution of type of diabetes, incidental findings and nodules, by CAC categories. (DOCX)

S2 Table Medical cares in patients with pulmonary nodules. (DOCX)

S3 Table Clinical characteristics of the 3 patients with lung cancers. (DOCX)

S4 Table Cares for other pulmonary incidental findings. (DOCX)

S5 Table Cares for non-pulmonary incidental findings. (DOCX)

S1 File Clinical data about patients with lung cancers. (DOCX)

## Abbreviations

BMI: Body Mass Index
CAC: Coronary Artery Calcium Score
CV: Cardiovascular
HTA: hypertension
LDCT: Low Dose Computed Tomography
MDCT: Multiple Detector Computed Tomography
PY: Pack-year
TDM: Tomodensitometry
TD1: type 1 diabetes
TD2: type 2 diabetes
TD3: type 3 diabetes
