# Supplementary material for: Higher prevalence of incidental findings identified upon coronary calcium score assessment in type 2 and type 3 diabetes versus type 1 diabetes
Source: PLoS One. 2021 May 24;16(5):e0251693. doi: 10.1371/journal.pone.0251693 (PMC8143389; doi:10.1371/journal.pone.0251693)
Supplement: S1 Table — (DOCX) [file pone.0251693.s001.docx]

**S1 Table: Distribution of type of diabetes, incidental findings and nodules, by CAC categories**.

| **CAC group (n=732)** | **< 100 (n=476)** | **100-400 (n=122)** | **> 400 (n=133)** | **p** |
| --- | --- | --- | --- | --- |
| **Type of diabetes** |  |  |  |  |
| **Type 1** | 128 (26.9) | 33 (27.0) | 26 (19.4) |  |
| **Type 2** | 308 (64.7) | 79 (64.8 ) | 95 (70.9 ) | 0.510 |
| **Type 3** | 40 (8.4) | 10 (8.2) | 13 (9.7) |  |
| **Incidentalomas** | 76 (16.0) | 16 (13.1) | 25 (18.7) | 0.482 |
| **Nodules** | 21 (4.4) | 4 (3.3) | 6 (4.5) | 0.847 |

Results are expressed as number (pourcentage).
