# Supplementary material for: Higher prevalence of incidental findings identified upon coronary calcium score assessment in type 2 and type 3 diabetes versus type 1 diabetes
Source: PLoS One. 2021 May 24;16(5):e0251693. doi: 10.1371/journal.pone.0251693 (PMC8143389; doi:10.1371/journal.pone.0251693)
Supplement: S2 Table — (DOCX) [file pone.0251693.s002.docx]

**S2 Table: Medical cares in patients with pulmonary nodules**

| n=31 | < 5mm  (n=12) | 5-10 mm  (n=11) | 10-30 mm  (n=6) | > 30 mm  (n=2) |
| --- | --- | --- | --- | --- |
| Diagnosis management | | | | |
| Specialized medical advice by phone | 7 | 9 | 6 | 2 |
| Tomodensitometry | 10 | 9 | 6 | 2 |
| PET tomography | 0 | 0 | 1 | 2 |
| No diagnostic management | 2 | 2 | 0 | 0 |
| Cancer | 0 | 0 | 1 | 2 |
| Therapeutic management | | | | |
| Referral to pneumologist | 5 | 6 | 6 | 2 |
| Monitored Only | 10 | 9 | 5 | 0 |
| Treatment | 0 | 0 | 1 | 2 |
