# Supplementary material for: Higher prevalence of incidental findings identified upon coronary calcium score assessment in type 2 and type 3 diabetes versus type 1 diabetes
Source: PLoS One. 2021 May 24;16(5):e0251693. doi: 10.1371/journal.pone.0251693 (PMC8143389; doi:10.1371/journal.pone.0251693)
Supplement: S3 Table — (DOCX) [file pone.0251693.s003.docx]

**S3 Table:** **Clinical characteristics of the 3 patients with lung cancers**

|  | **Patient 1** | **Patient 2** | **Patient 3** |
| --- | --- | --- | --- |
| Age (years) | 64 | 55 | 66 |
| Sex | M | M | M |
| BMI (kg/m^2^) | 26.5 | 38 | 28 |
| Diabetes evolution (years) | 4 | 16 | 32 |
| Type of diabetes | TD2 | TD2 | TD2 |
| HBA1c (%) | 6.4 | 8.2 | 6.6 |
| Insulin therapy | No | No | Yes |
| CAC (AU) | 228 | 0 | 0 |
| Smoking | yes | yes | yes |
| Smocking (Number of PY) | >20 | >20 | 10-20 |
| HTA | yes | yes | yes |
| Microalbuminuria | No | No | Yes |
| Renal Failure | No | No | Yes |
| Retinopathy | No | Yes | Yes |

M: male, PY: pack-years, TD2: type 2 diabetes

**S2 Table**: Cares for other pulmonary incidentalomas

|  | **Specialized medical advices** | **TDM** | **Referral to pneumologist** | **Treatment / diagnosis** | **NTA** |
| --- | --- | --- | --- | --- | --- |
| **Emphysema (n=8)** | 4^a^ | 4 | 1 | 1 (β2 mimetics) | 1 |
| **Pleural diseases (=7)** | 6^b^ | 4 | 4 | - | 1 |
| **Bronchitis (n=4)^c^** | - | 1 | 1 | 1 (β2 mimetics) | 0 |
| **Bronchiectasis (n=4)** | 2 | 2 | 1 | - | 2 |
| **Pneumonia (n=4)^d^** | - | - | 2 | 2 (antibiotics) | 0 |
| **Hilar Lymphadenopathies (n=18)** | 5 |  | 2 | 1 DIP  1 pneumoconiosis | 13^f^ |

^a^ 3 others patients had chronic obstructive bronchitis already followed by a pneumologist and not initially reported by the patient.

^b^ 1 subject with calcified pleural plaques, 2 subjects with pleural cysts and 3 subjects with pleural effusion

^c^ 3/4 patients were asymptomatic

^d^ 2 asymptomatic patients

^f^ not investigated due to small size, absence of inflammatory syndrome or blood count abnormalities

DIP: diffuse interstitial pneumonia; NTA: not taken into account

**S3 Table:** Cares for non-pulmonary incidentalomas

| **Type of incidental findings** | **Specialized medical**  **advices** | **Others exams** | **Referral to a specialist** | **Treatment / diagnosis** | **NTA** |
| --- | --- | --- | --- | --- | --- |
| **Heart (n=13)** |  |  |  |  |  |
| **- 11 pericardial infusion (3 minor)** | 8 | 6a | 6 | No treatment | 0 |
| **- 2 pulmonary hypertension** | - | 1a | No |  | 1 |
| **Vascular (n=9)** |  |  |  |  |  |
| **- 7 dilated thoracic aorta** | 4 | 1 TDM | 4 | No surgery  / 3 follow-up | 0 |
| **- 2 splenic aneurysms** | 2 | 1 TDM | 2 | 2 follow-up |  |
| **Thymic (n=5)** |  |  |  | No malignancy |  |
| **- 4 thymic residuals** | 5 | 4 TDM | 5 (thoracic  surgeon) | No surgery  3 patients | 0 |
| **- 1 thymic cyst** |  | 1PET TDM |  | monitored |  |
| **Digestive (n=4)** |  |  |  |  |  |
|  |  |  |  | gallstone |  |
| **- 1 dilated bile ducts** | 1 | 1 TDM | 1 | extraction | 0 |
| **- 2 hiatal hernia** | - | - | - | - |  |
| **- 1 liver cyst** | 1 | - | - | - |  |
| **Vertebral hemangioma (n=1)** | - | - | - | - | 0 |
