# Supplementary material for: Higher prevalence of incidental findings identified upon coronary calcium score assessment in type 2 and type 3 diabetes versus type 1 diabetes
Source: PLoS One. 2021 May 24;16(5):e0251693. doi: 10.1371/journal.pone.0251693 (PMC8143389; doi:10.1371/journal.pone.0251693)
Supplement: S4 Table — (DOCX) [file pone.0251693.s004.docx]

**S4 Table**: **Cares for other pulmonary incidental findings**

|  | **Specialized medical advices** | **TDM** | **Referral to pneumologist** | **Treatment / diagnosis** | **NTA** |
| --- | --- | --- | --- | --- | --- |
| **Emphysema (n=8)** | 4^a^ | 4 | 1 | 1 (β2 mimetics) | 1 |
| **Pleural diseases (=7)** | 6^b^ | 4 | 4 | - | 1 |
| **Bronchitis (n=4)^c^** | - | 1 | 1 | 1 (β2 mimetics) | 0 |
| **Bronchiectasis (n=4)** | 2 | 2 | 1 | - | 2 |
| **Pneumonia (n=4)^d^** | - | - | 2 | 2 (antibiotics) | 0 |
| **Hilar Lymphadenopathies (n=18)** | 5 |  | 2 | 1 DIP  1 pneumoconiosis | 13^f^ |

^a^ 3 others patients had chronic obstructive bronchitis already followed by a pneumologist and not initially reported by the patient.

^b^ 1 subject with calcified pleural plaques, 2 subjects with pleural cysts and 3 subjects with pleural effusion

^c^ 3/4 patients were asymptomatic

^d^ 2 asymptomatic patients

^f^ not investigated due to small size, absence of inflammatory syndrome or blood count abnormalities

DIP: diffuse interstitial pneumonia; NTA: not taken into account
