# Supplementary material for: Higher prevalence of incidental findings identified upon coronary calcium score assessment in type 2 and type 3 diabetes versus type 1 diabetes
Source: PLoS One. 2021 May 24;16(5):e0251693. doi: 10.1371/journal.pone.0251693 (PMC8143389; doi:10.1371/journal.pone.0251693)
Supplement: S5 Table — (DOCX) [file pone.0251693.s005.docx]

**S5 Table**: **Cares for non-pulmonary incidental findings**

| **Type of incidental findings** | **Specialized medical**  **advices** | **Others exams** | **Referral to a specialist** | **Treatment / diagnosis** | **NTA** |
| --- | --- | --- | --- | --- | --- |
| **Heart (n=13)** |  |  |  |  |  |
| **- 11 pericardial infusion (3 minor)** | 8 | 6^a^ | 6 | No treatment | 0 |
| **- 2 pulmonary hypertension** | - | 1^a^ | No |  | 1 |
| **Vascular (n=9)** |  |  |  |  |  |
| **- 7 dilated thoracic aorta** | 4 | 1 TDM | 4 | No surgery  / 3 follow-up | 0 |
| **- 2 splenic aneurysms** | 2 | 1 TDM | 2 | 2 follow-up | 0 |
| **Thymic (n=5)** |  |  |  | No malignancy |  |
| **- 4 thymic residuals** | 5 | 4 TDM | 5 (thoracic  surgeon) | No surgery  3 patients | 0 |
| **- 1 thymic cyst** |  | 1PET TDM |  | monitored |  |
| **Digestive (n=4)** |  |  |  |  |  |
|  |  |  |  | gallstone |  |
| **- 1 dilated bile ducts** | 1 | 1 TDM | 1 | extraction | 0 |
| **- 2 hiatal hernia** | - | - | - | - |  |
| **- 1 liver cyst** | 1 | - | - | - |  |
| **Vertebral hemangioma (n=1)** | - | - | - | - | 0 |

NTA : not taken into acount

^a^ cardiac echographies
