# Supplementary material for: Higher prevalence of incidental findings identified upon coronary calcium score assessment in type 2 and type 3 diabetes versus type 1 diabetes
Source: PLoS One. 2021 May 24;16(5):e0251693. doi: 10.1371/journal.pone.0251693 (PMC8143389; doi:10.1371/journal.pone.0251693)
Supplement: S1 File — (DOCX) [file pone.0251693.s006.docx]

**S1 File : Clinical data about patients with lung cancers**

Three patients with nodules (all above 30 mm) had pulmonary carcinoma, 2 of them being initially metastatic. Clinical characteristics of subjects with lung cancers are detailed in the S1 table. All were active smokers. Chemotherapy and immunotherapy were managed by oncologists and pneumologists. Two patients had lesions in progress at 2 and 3 years of follow up and one was stable at 3 years of follow up.
